# Assessment of naive indolent lymphoma using whole-body diffusion-weighted imaging and T2-weighted MRI: results of a prospective study in 30 patients

**DOI:** 10.1186/s40644-020-00371-6

**Published:** 2021-01-07

**Authors:** Gil-Sun Hong, Eun Jin Chae, Jin-Sook Ryu, Sun Young Chae, Hyo Sang Lee, Dok Hyun Yoon, Cheolwon Suh

**Affiliations:** 1 Department of Radiology and Research Institute of Radiology, University of Ulsan College of Medicine, Asan Medical Center, 88, Olympic-ro 43-gil, Songpa-gu, Seoul, South Korea; 2 Department of Nuclear Medicine, University of Ulsan College of Medicine, Asan Medical Center, 88, Olympic-ro 43-gil, Songpa-gu, Seoul, South Korea; 3 Department of Nuclear Medicine, University of Ulsan College of Medicine, Gangneung Asan Hospital, 38, Bangdong-gil, Sacheon-myeon, Gangneung, South Korea; 4 Department of Oncology, University of Ulsan College of Medicine, Asan Medical Center, 88, Olympic-ro 43-gil, Songpa-gu, Seoul, South Korea

**Keywords:** Indolent lymphoma, Whole-body magnetic resonance imaging, Diffusion-weighted imaging with background body signal suppression, T2-weighted short-tau inversion recovery MRI

## Abstract

**Background:**

We prospectively evaluated the diagnostic utility of whole-body diffusion-weighted imaging with background body signal suppression and T2-weighted short-tau inversion recovery MRI (WB-DWIBS/STIR) for the pretherapeutic staging of indolent lymphoma in 30 patients.

**Methods:**

This prospective study included 30 treatment-naive patients with indolent lymphomas who underwent WB-DWIBS/STIR and conventional imaging workup plus biopsy. The pretherapeutic staging agreement, sensitivity, and specificity of WB-DWIBS/STIR were investigated with reference to the multimodality and multidisciplinary consensus review for nodal and extranodal lesions excluding bone marrow.

**Results:**

In the pretherapeutic staging, WB-DWIBS/STIR showed very good agreement (κ = 0.96; confidence interval [CI], 0.88–1.00), high sensitivity (93.4–95.1%), and high specificity (99.0–99.4%) for the whole-body regions. These results were similar to those of ^18^F-FDG-PET/CT, except for the sensitivity for extranodal lesions. For extranodal lesions, WB-DWIBS/STIR showed higher sensitivity compared to ^18^F-FDG-PET/CT for the whole-body regions (94.9–96.8% vs. 79.6–86.3%, *P* = 0.058).

**Conclusion:**

WB-DWIBS/STIR is an effective modality for the pretherapeutic staging of indolent lymphoma, and it has benefits when evaluating extranodal lesions, compared with ^18^F-FDG-PET/CT.

## Background

Recently, the “watchful waiting” strategy has become a treatment option in indolent non-Hodgkin’s lymphoma, particularly considering its indolent clinical behavior with a prolonged natural history. This suggests the need for precise initial staging followed by strict monitoring using imaging examinations [1]. 18F-fluorodeoxyglucose-positron emission tomography/computed tomography (^18^F-FDG-PET/CT) has only a limited role for the non-FDG-avid subtype of lymphoma, as it is based on increased glucose metabolism. Nevertheless, in clinical practice, many physicians use ^18^F-FDG-PET/CT as the standard imaging modality for the staging of indolent lymphoma and evaluation of FDG-avid lymphoma. Although CT has been recommended for the monitoring and imaging follow-up of non-FDG-avid histotypes of lymphoma [2–6], both ^18^F-FDG-PET/CT and CT present a risk of secondary cancer due to radiation exposure. The risk is increased when several scans are performed at follow-ups and is of particular concern for young patients. Furthermore, the use of contrast agents is limited in patients with abnormal kidney function [7].

The role of whole-body magnetic resonance imaging (WB-MRI) has been well established in hematologic malignancies and multiple metastases, especially with regard to the evaluation of bone involvement in these diseases [8–15]. However, relatively little information exists on its utility for the evaluation of indolent lymphoma. Moreover, there is still debate over the value of WB-MRI for evaluating the bone marrow involvement in indolent lymphoma. Prior studies reported that WB-MRI has low sensitivity for the detection of low-volume bone marrow involvement in the indolent lymphoma [9, 16]. As a result, the bone marrow biopsy is routinely recommended in non-Hodgkin’s lymphoma, while it is no longer required for Hodgkin’s lymphoma [1]. Therefore, the requirement of studying multiple sequences for staging naive indolent lymphoma is questionable.

In the present study, we aimed to determine the diagnostic value of WB-MRI (WB-DWIBS/STIR), which only comprises diffusion-weighted imaging with background body signal suppression (DWIBS) and T2-weighted short-tau inversion recovery (T2-STIR), for pretherapeutic staging of indolent lymphoma.

## Methods

### Study design and population

Thirty adult patients histologically diagnosed with indolent non-Hodgkin’s lymphoma at the department of hemato-oncology between June 20, 2013, and April 30, 2015, were consecutively enrolled. The inclusion criteria were 1) older than 19 years; 2) diagnosis of a histologic type of indolent non-Hodgkin’s lymphoma; and 3) no prior treatment for the lymphoma. The exclusion criterion was a patient who was unable or unwilling to undergo MRI. In addition to the conventional workup with ^18^F-FDG-PET/CT, contrast-enhanced whole-body CT, and biopsy, the patients underwent WB-DWIBS/STIR. The lymphomas were classified into histologic subtypes according to the 2016 WHO classification for lymphoid malignancy. The median time interval between the lymphoma biopsy and the WB-DWIBS/STIR acquisition was 23 days (range, 7–945 days).

### MRI techniques

WB-MRI was performed using a 3-T scanner (Ingenia; Philips Healthcare, Best, The Netherlands) with parallel radiofrequency transmission and phased-array surface coils. The protocol comprised DWIBS (b-factors = 0 and 1000 s/mm2) and T2-STIR sequences. Briefly, the scan parameters for DWIBS included the following: repetition time/echo time/inversion recovery, 8897/85/250 ms (for the head and neck) and 8619/89/250 ms (for the body); flip angle 90°; matrix 184 × 113 (for the head and neck) and 92 × 242 (for the body); field of view 550 × 344 mm (for the head and neck) and 200 × 518 mm (for the body); and slice thickness 3 mm (for the head and neck) and 5 mm (for the body). Parameters for T2-STIR included the following: repetition time/echo time/inversion recovery, 5510/76/230 ms (for the head and neck) and 6659/60/220 ms (from head to lower limbs); flip angle, 60°; matrix, 560 × 206 (for the head and neck) and 408 × 206 (from head to lower limbs); field of view, 550 × 299 mm (for the head and neck) and 550 × 371 mm (from the head to the lower limbs); and slice thickness, 3 mm (for the head and neck) and 5 mm (from the head to lower limbs). DWIBS with free-breathing was acquired in the transverse plane for the head and neck, reformatted to coronal images, and acquired in the coronal plane for the region from the thorax to the lower limbs. Whole-body DWIBS images were acquired over a total scan time of 17–18 min and comprised five stacks. T2-STIR images with respiratory triggering were acquired in the transverse plane for the head and neck and coronal plane from the head to lower limbs, with five stacks. Whole-body T2-STIR images were acquired over a total scan time of 9–10 min. The total acquisition time for WB-MRI was no more than 26–28 min. Each of the coronal whole-body T2-STIR images and DWIBS was created by merging these five stacks using software implemented in the standard operating console of the scanner. Maximum intensity projection images of DWIBS were then reconstructed.

### WB-DWIBS/STIR image analysis

All images were reviewed using a local PACS monitor and DICOM image-viewing software (PetaVision; Asan Medical Center). Two board-certified radiologists (observer 1, G.S.H.; and observer 2, E.J.C.) who were blinded to ^18^F-FDG-PET/CT data and all clinical information, except for the diagnosis of lymphoma (although they were unaware of the type or grade of lymphoma) independently analyzed WB-DWIBS/STIR in a visual manner and evaluated the presence/absence of lymphoma in each region. The body was divided into nodal regions and extranodal sites. Nodal regions were determined by reference to guidelines (with modification) for delineating the involved fields of nodal sites: the neck, supraclavicular regions, mediastinum, hilar regions, axilla, abdominal paraaortic region, mesentery, pelvic iliac regions, and inguinal regions [17]. If a positive nodal lesion was found at a location other than those mentioned above, each of the observers described the unique location of the lesion (e.g., internal mammary node, cardiophrenic angle, hepatoduodenal ligament) and assigned it as “other nodal regions” for the statistical analysis. The lymphoma stage was evaluated according to the modified Ann Arbor classification system. Stage 0 was defined as the observer finding of no positive lesions.

The criteria for a positive lesion on WB-DWIBS/STIR were set based on previous studies [11, 18, 19]. Morphological features of lymph nodes, such as loss of fat hilum, heterogeneity, and a short-axis diameter of at least 1 cm were used as diagnostic criteria. On T2-STIR, extranodal sites were considered positive according to the following criteria: focal or diffuse abnormal signal intensity (relatively to the surrounding tissue) in the spleen, salivary glands, or gastrointestinal tract; nodular infiltration in the lung; soft tissue infiltration with abnormal signal intensity (relative to the surrounding tissue) in other extranodal sites; splenomegaly (more than 13 cm in maximal size); and asymmetrical enlargement of bilateral organs, such as the lacrimal glands, salivary glands, and tonsils. On DWIBS, focally increased signal intensity in extranodal organs (spleen, gallbladder, adrenal glands, prostate, testis, penis, endometrium, ovaries, brain, peripheral nerve, spinal cord, salivary glands, tonsils, and bone marrow) was considered positive for lymphoma involvement, as these organs are known to show impeded diffusion in the normal state [20, 21]. Apparent diffusion coefficient (ADC) measurements were not used for the characterization of lymph nodes or extranodal lesions because there are no validated ADC criteria.

### PET/CT image analysis

Apart from the process performed in the clinical practice, the PET/CTs were retrospectively reviewed. For this purpose, two board-certified nuclear medicine physicians (observer 3, S.Y.C.; and observer 4, H.S.L.) who were blinded to the patients’ clinical information independently analyzed ^18^F-FDG-PET/CT in a visual manner with reference to the diagnostic criteria used in previous studies [22–24]. In the evaluation of ^18^F-FDG-PET/CT, non-contrast-enhanced CT was used to identify the anatomical locations of positive lesions and to identify whether or not lesions were distinct from surrounding tissue, including those lesions with low FDG avidity. The standardized uptake values of each positive lesion were not recorded or used as a diagnostic criterion because there are no validated standardized uptake value criteria for the non-FDG-avid subtype of lymphoma. A lesion was regarded as positive if partial or diffuse FDG uptake was higher than the surrounding tissue activity or if there was activity at a location where no physiologic tracer uptake should have occurred. For the spleen, tracer uptake higher than that of the liver was judged as positive.

### Reference standard

According to previous studies [8, 25–27], the multimodality and multidisciplinary consensus review can be used as a standard for studies on diagnostic performance wherein an independent reference standard does not exist. Reference data included full knowledge of all initial and follow-up investigations (follow-up duration [mean ± standard deviation], 116 ± 65 days). In this period, the presence and absence of disease were determined using all available clinical data, including results of initial imaging, clinical staging, follow-up imaging, histopathology (i.e., biopsy), and patients’ clinical course. In the absence of histological evidence, the disease was assumed to be positive if there was multimodality consensus (among contrast-enhanced CT, ^18^F-FDG-PET/CT, and WB-DWIBS/STIR) or if the pre-existing lesions had progressed or responded to therapy in follow-up images. Compared to WB-MRI acquisition, the time interval (mean ± standard deviation) was 5.57 ± 5.97 days for PET-CT, 7.90 ± 9.23 days for contrast-enhanced CT, and 116.19 ± 65.49 days for the last follow-up imaging (contrast-enhanced CT or MRI).

### Statistical analysis

Staging agreement and diagnostic performance (region-based sensitivity and specificity) were calculated for whole-body regions excluding the bone marrow. To illustrate the advantage of WB-DWIBS/STIR compared to PET/CT, the staging agreement and region-based sensitivity and specificity of conventional PET/CT imaging were also evaluated. Generalized estimation equations were used for sensitivity and specificity comparisons to account for multiple regions per patient. Cohen’s kappa statistic was used to calculate the agreement between the WB-DWIBS/STIR and the reference standard for different sites. For each subtype of indolent lymphoma (i.e., follicular lymphoma and MALT lymphoma), region-based sensitivity and specificity were calculated for WB-DWIBS/STIR and ^18^F-FDG-PET/CT. All statistical analyses were performed using SAS version 9.4 (SAS Institute; Cary, NC). Two-tailed significance thresholds of *P* <  0.05 were used.

## Results

### Patient characteristics

Table 1 summarizes the patient characteristics. The study cohort included patients with 14 follicular lymphomas (type I and II), 14 extranodal marginal zone lymphomas of mucosa-associated lymphoid tissue (MALToma), one chronic lymphocytic leukemia/small lymphocytic lymphoma (CLL/SLL), and one nodal marginal zone lymphoma. The study population comprised 10 patients in stage IE, five in stage II, seven in stage III, two in stage IIIS, one with stage IIIE, and five with stage IV. In the 30 patients, a total of 98 nodal regions and 23 non-bone marrow extranodal sites were positive according to the reference standard. Of these, 18 nodal regions and 16 extranodal sites were histologically diagnosed with lymphoma involvement following a biopsy. Of the remaining 80 nodal regions and seven extranodal sites, 72 nodal regions and six extranodal sites were considered to be positive according to the consensus of the four observers plus the image review of contrast-enhanced CT. The remaining eight nodal regions (two paraaortic regions, two hilar regions, one axilla, one cardiophrenic angle, one hepatoduodenal ligament, and one mesenteric region) and one extranodal lesion (skin, subcutaneous layer, and/or muscles) were confirmed to have lymphoma involvement on the basis of the post-treatment response on follow-up CT. The 23 positive extranodal sites included the orbit (*n* = 9); salivary gland (*n* = 4); tonsil (*n* = 1); lung (*n* = 1); spleen (*n* = 3); stomach (*n* = 2); and skin, subcutaneous layer, and/or muscles (*n* = 3).

**Table 1:** Patient Characteristics

Characteristics	
No. of patients *	30
Sex *	
Men	23
Women	7
Age (years)	
Mean ± SD	55.5 ± 14.2
Range	26–82
Pathologic subtype*	
Follicular lymphoma type I and II	14
Extranodal marginal zone lymphoma of mucosa-associated lymphoid tissue	14
Chronic lymphocytic leukemia/small lymphocytic lymphoma	1
Nodal marginal zone lymphoma	1
Pretherapeutic staging based on the reference standard*^§^	
IE	10
II	5
III	7
IIIS	2
IIIE	1
IV	5

^§^The pretherapeutic staging does not reflect bone marrow involvement of lymphoma

*Data are numbers of patients

### Diagnostic performance

Table 2 summarizes the agreement of WB-DWIBS/STIR and ^18^F-FDG-PET/CT for the pretherapeutic staging in comparison with the reference standard. For the pretherapeutic staging of patients with indolent lymphoma, WB-DWIBS/STIR showed very good agreement (κ = 0.96; CI, 0.88–1.00) with the reference standard. ^18^F-FDG-PET/CT showed similar agreement as WB-DWIBS/STIR.

**Table 2:** Pretherapeutic Staging of 30 Patients with Indolent Lymphoma using WB-DWIBS/STIR and ^18^F-FDG-PET/CT

		Reference Staging*						
		**Stage IE**	**Stage II**	**Stage III**	**Stage IIIS**	**Stage IIIE**	**Stage IV**	**Kappa [95% CI]** ^**§**^
**WB-DWIBS/STIR (R1)**	Stage IE	10	0	0	0	0	0	0.96 [0.88–1.00]
	Stage II	0	5	0	0	0	0	
	Stage III	0	0	6	0	0	0	
	Stage IIIS	0	0	1	2	0	0	
	Stage IIIE	0	0	0	0	1	0	
	Stage IV	0	0	0	0	0	5	
**WB-DWIBS/STIR (R2)**	Stage IE	10	0	0	0	0	0	0.96 [0.88–1.00]
	Stage II	0	5	0	0	0	0	
	Stage III	0	0	6	0	0	0	
	Stage IIIS	0	0	1	2	0	0	
	Stage IIIE	0	0	0	0	1	0	
	Stage IV	0	0	0	0	0	5	
^**18**^**F-FDG-PET/CT (R1)**	Stage 0	1	0	0	0	0	0	0.92 [0.80–1.00]
	Stage IE	9	0	0	0	0	0	
	Stage II	0	4	0	0	0	0	
	Stage III	0	0	7	0	0	0	
	Stage IIIS	0	1	0	2	0	0	
	Stage IIIE	0	0	0	0	1	0	
	Stage IV	0	0	0	0	0	5	
^**18**^**F-FDG-PET/CT (R2)**	Stage IE	10	0	0	0	0	0	0.96[0.88–1.00]
	Stage II	0	4	0	0	0	0	
	Stage III	0	0	7	0	0	0	
	Stage IIIS	0	1	0	2	0	0	
	Stage IIIE	0	0	0	0	1	0	
	Stage IV	0	0	0	0	0	5	

^§^The pretherapeutic staging does not reflect bone marrow involvement of lymphoma

*Data are numbers of patients. *R1* = observer 1, *R2* = observer 2, *WB-DWIBS/STIR* = whole-body diffusion-weighted imaging with background body signal suppression and T2-weighted short-tau inversion recovery magnetic resonance imaging, ^*18*^*F-FDG-PET/CT* = 18F-fluorodeoxyglucose-positron emission tomography-computed tomography

**Fig. 1:**
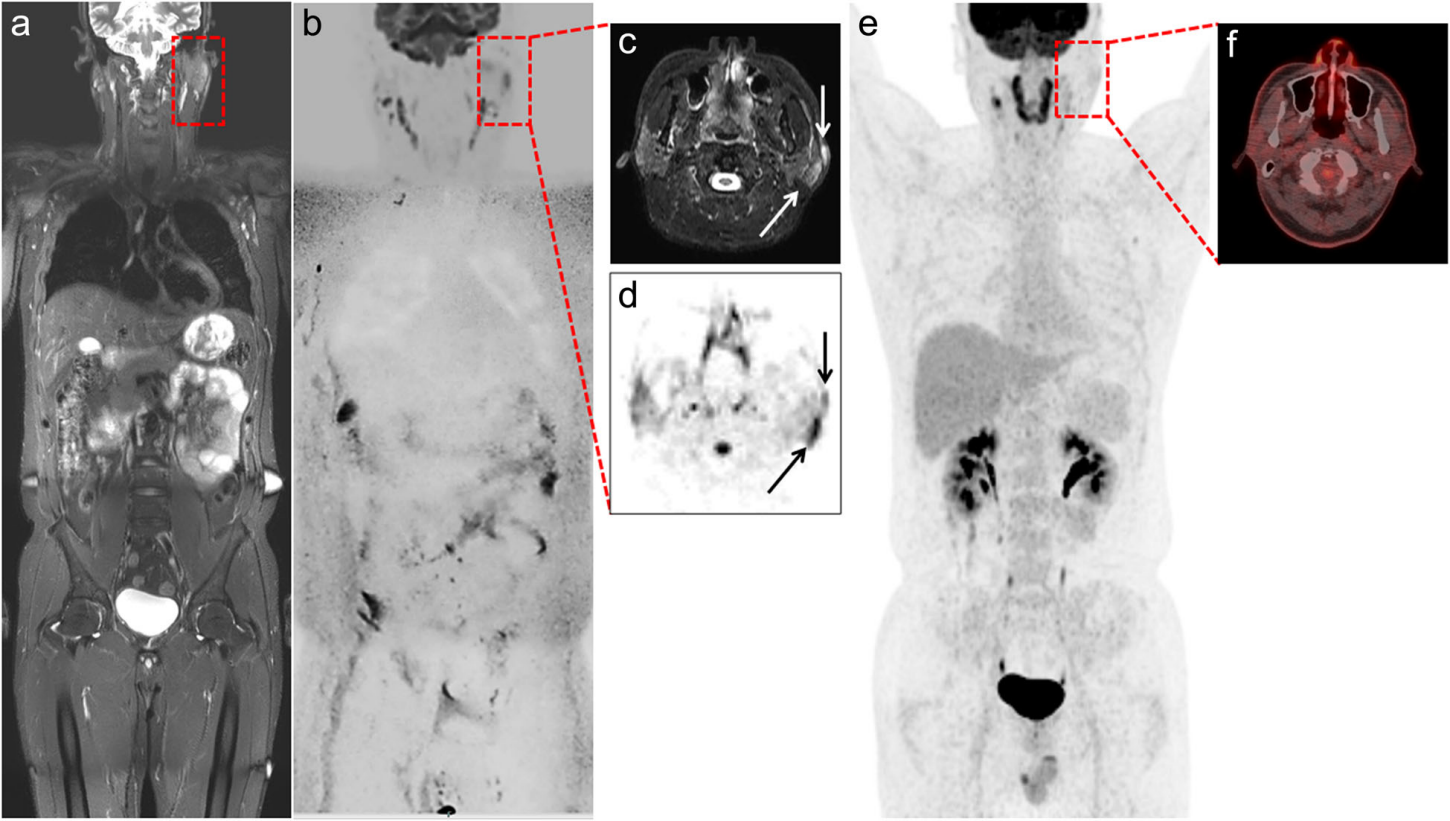
A 43-year-old man with MALToma of pretherapeutic stage IV. **a** Coronal whole-body T2-STIR, **b** DWIBS, **c** axial T2-STIR, **d** and axial DWIBS show lymphoma involvement in the left parotid gland and overlying skin (dashed square and arrows). **e** Coronal whole-body ^18^F-FDG-PET/CT MIP and (f) ^18^F-FDG-PET/CT axial fusion images do not show 18F-FDG uptake in the corresponding area. DWIBS = diffusion-weighted imaging with background body signal suppression, T2-STIR = T2-weighted short-tau inversion recovery, ^18^F-FDG-PET/CT = 18F-fluorodeoxyglucose-positron emission tomography-computed tomography, MIP = maximum intensity projection

**Fig. 2:**
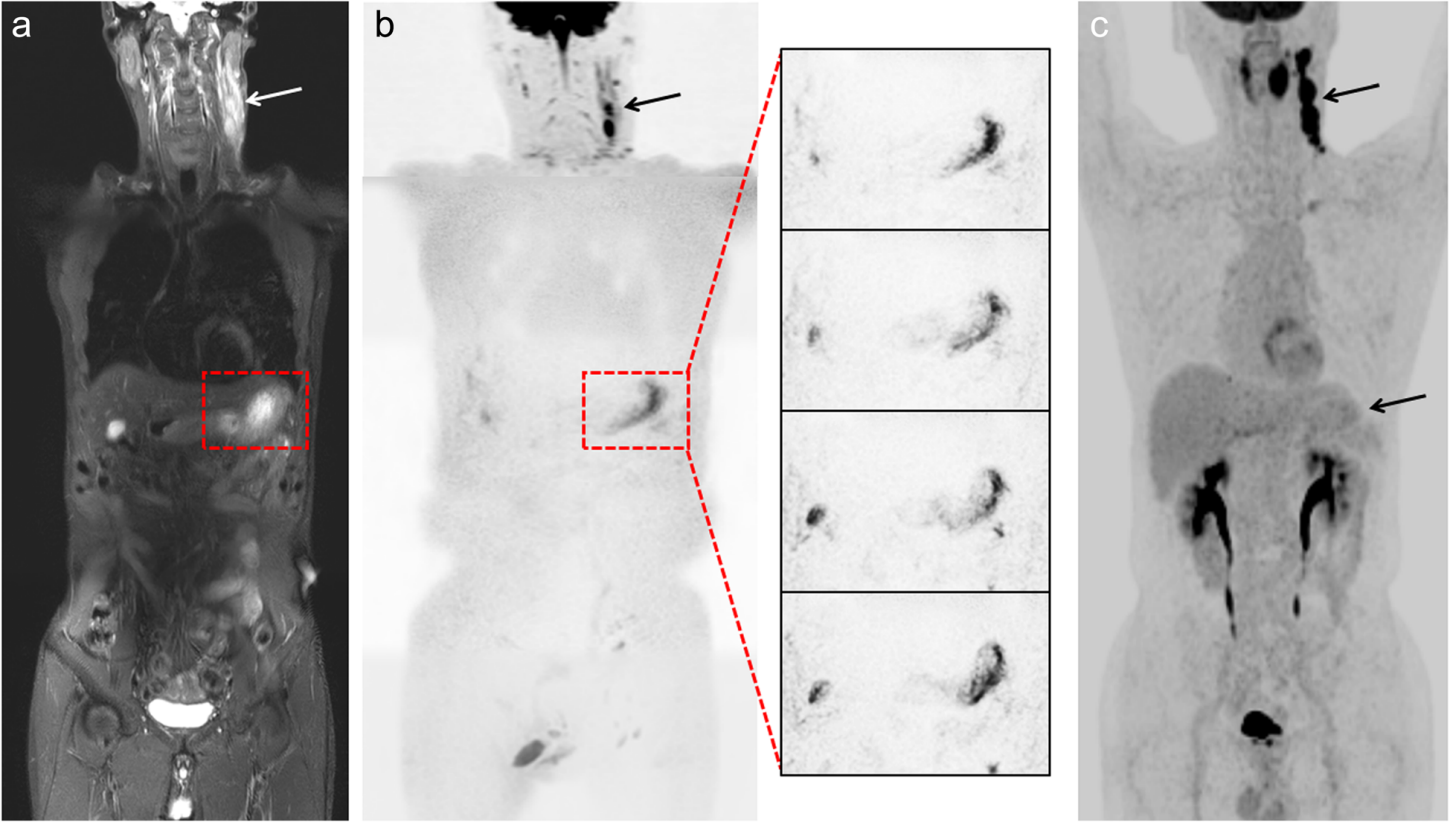
A 59-year-old man with MALToma of pretherapeutic stage IV. **a** Coronal whole-body T2-STIR and **b** DWIBS show lymphoma involvement of the stomach (dashed square) and lymph nodes in the neck (arrow). Magnified DWIBS images show high signal intensity along the greater curvature of the gastric body. **c** Coronal whole-body ^18^F-FDG-PET/CT MIP do not show 18F-FDG uptake in the corresponding area of stomach. DWIBS = diffusion-weighted imaging with background body signal suppression, T2-STIR = T2-weighted short-tau inversion recovery, ^18^F-FDG-PET/CT = 18F-fluorodeoxyglucose-positron emission tomography-computed tomography, MIP = maximum intensity projection

Table 3 summarizes the region-based sensitivities and specificities of WB-DWIBS/STIR and ^18^F-FDG-PET/CT with respect to the reference standard. WB-DWIBS/STIR showed a high sensitivity (93.4–95.1%) and specificity (99.0–99.4%). For extranodal lesions, WB-DWIBS/STIR a higher sensitivity compared to ^18^F-FDG-PET/CT (94.9–96.8% vs. 79.6–86.3%, *P* = 0.058). Figures 1 and 2 show representative cases of indolent lymphoma involving extranodal lesions. In the regional detail analysis (Table 4), WB-DWIBS/STIR demonstrated limitations for evaluating some thoracic nodal regions (hilar region [κ = 0.78]), other nodal regions (internal mammary region, cardiophrenic angle, intercostal area, and hepatoduodenal ligaments [κ = 0.85–0.89]), and one extranodal lesion (stomach [κ = 0.65]).

**Table 3:** Region-based sensitivities and specificities for WB-DWIBS/STIR and ^18^F-FDG-PET/CT

	Overall	***P***	Nodal	***P***	Extranodal	***P***
**Sensitivity**^**§**^						
WB-DWIBS/STIR (R1)	95.1	0.117	95.4	0.511	94.9	0.058
WB-DWIBS/STIR (R2)	93.4		93.0		96.8	
^18^F-FDG-PET/CT (R1)	91.8		94.1		79.6	
^18^F-FDG-PET/CT (R2)	89.3		91.1		86.3	
**Specificity**^**§**^						
WB-DWIBS/STIR (R1)	99.4	< 0.001	99.3	< 0.001	99.5	0.439
WB-DWIBS/STIR (R2)	99.0		98.8		99.3	
^18^F-FDG-PET/CT (R1)	97.3		95.4		98.9	
^18^F-FDG-PET/CT (R2)	95.6		92.1		98.6	

^**§**^Data does not reflect bone marrow involvement of lymphoma. Data are shown as percentages. Generalized estimating equations were used for sensitivity and specificity comparisons to allow multiple regions per patient to be taken into account. *R1* observer 1, *R2* observer 2, *WB-DWIBS/STIR* whole-body diffusion-weighted imaging with background body signal suppression and T2-weighted short-tau inversion recovery magnetic resonance imaging, ^*18*^*F-FDG-PET/CT* = 18F-fluorodeoxyglucose-positron emission tomography-computed tomography

**Table 4:** Agreement between WB-DWIBS/STIR and the reference standard for different sites

Parameter	Kappa values	
	WB-DWIBS/STIR (R1)	WB-DWIBS/STIR (R2)
**Nodal**	0.96	0.93
Cervical	0.93	0.93
Axillary	1.00	0.93
Mediastinal	1.00	1.00
Hilar	0.78	0.78
Paraaortic	0.93	0.93
Mesenteric	1.00	1.00
Pelvic	1.00	1.00
Inguinal	0.93	0.93
Others	0.89	0.85
**Extranodal**	0.71	0.74
Orbit	1.00	0.92
Salivary gland	1.00	1.00
Tonsil	1.00	1.00
Lung	1.00	1.00
Spleen	0.84	0.84
Stomach	0.65	0.65
Skin, subcutaneous layer and/or muscles	1.00	1.00

*R1* observer 1, *R2* observer 2, *WB-DWIBS/STIR* whole-body diffusion-weighted imaging with background body signal suppression and T2-weighted short-tau inversion recovery magnetic resonance imaging

**Fig. 3:**
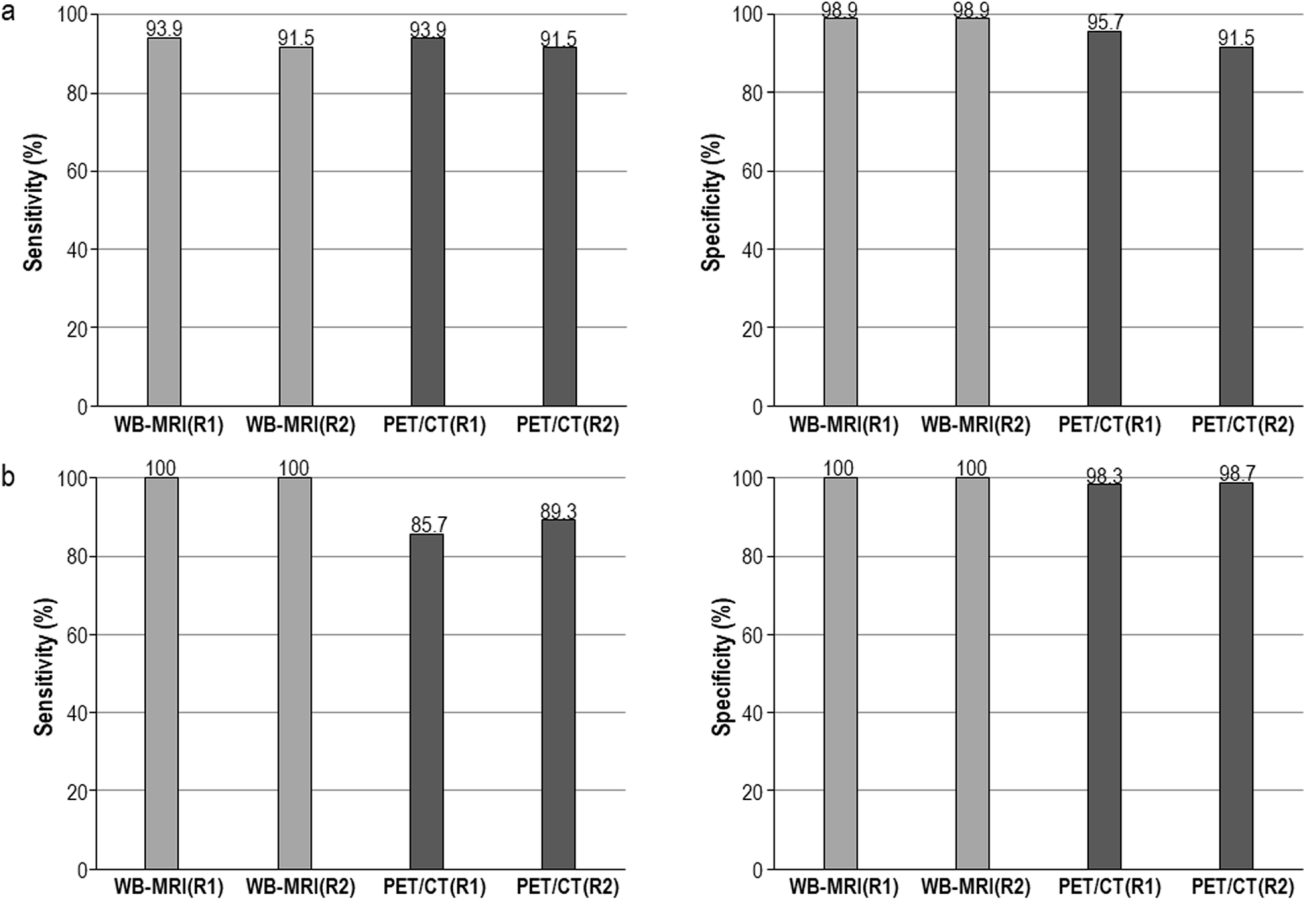
Region-based sensitivity and specificity in follicular lymphoma **a** and MALToma **b** for WB-DWIBS/STIR and ^18^F-FDG-PET/CT. WB-MRI = whole-body diffusion-weighted imaging with background body signal suppression and T2-weighted short-tau inversion recovery magnetic resonance imaging, PET/CT = 18F-fluorodeoxyglucose-positron emission tomography-computed tomography, R1 = observer 1, R2 = observer 2

When analyzed according to the histologic subtypes of lymphoma, WB-DWIBS/STIR for the pretherapeutic staging agreed with the reference standard in 100% of cases in both the follicular lymphoma and MALToma groups. WB-DWIBS/STIR showed sensitivities and specificities of 91.5–93.9% and 98.9%, respectively, for the follicular lymphoma group and 100 and 100%, respectively, for the MALToma group (Fig. 3).

## Discussion

The diagnostic value of WB-MRI has been established in the evaluation of solid tumors, such as lung cancer, hematologic malignancy (multiple myeloma and aggressive lymphoma), and bone metastasis, compared to ^18^F-FDG-PET/CT [9–15, 28]. With regard to evaluating and monitoring indolent lymphoma, WB-MRI has been considered a reasonable option under the watchful waiting strategy. However, evidence for the utility of WB-MRI for evaluating indolent lymphoma is relatively lacking. Our study clarifies that WB-DWIBS/STIR, which includes only essential sequences, is sufficiently effective for the initial workup of patients with indolent lymphoma.

The principal finding of this study was that WB-DWIBS/STIR performed excellently for pretherapeutic staging of indolent lymphomas. Most previous studies, consisting of patients with mixed subtypes of a relatively large number of aggressive lymphomas and some indolent lymphomas, investigated the staging accuracy of WB-MRI for each histologic lymphoma subtype [18, 19, 29–32]. However, except for one study, these studies included fewer than a dozen (5–13 patients) indolent lymphoma cases. Despite the small number of patients, to best our knowledge, our study comprised the second-largest number of indolent lymphoma cases among the studies covering both WB-MRI and ^18^F-FDG-PET/CT. Among 78 indolent lymphoma cases with variable FDG avidity of 140 lymphoma cases, Mayerhoefer et al. demonstrated that WB-MRI had a high staging accuracy (94.0%), which was much higher than that of ^18^F-FDG-PET/CT [18]. Stecco et al. reported an 85% accuracy of WB-MRI for staging gastrointestinal lymphomas among 17 patients with lymphoma (including 13 indolent lymphomas); they also demonstrated excellent agreement between WB-MRI and ^18^F-FDG-PET/CT [32]. In a meta-analysis for the staging accuracy of WB-MRI for indolent lymphomas, the pooled staging accuracy of WB-MRI was 96%, whereas that of ^18^F-FDG-PET/CT was 87% [33]. The high staging accuracy of WB-MRI in our study is consistent with the findings of Mayerhoefer et al. and the meta-analysis. However, there was no significant difference in the staging accuracy between WB-MRI and ^18^F-FDG-PET/CT in the present study. In subgroup analyses of indolent lymphomas, the meta-analytic staging accuracy of WB-MRI was 99% in follicular lymphoma and nodal marginal zone lymphoma, and 98% in CLL/SLL and MALToma. These results for WB-MRI are consistent with ours in both the follicular lymphoma and MALToma groups (100%, respectively). In agreement with previous studies, our study shows that WB-MRI is highly accurate in the staging of naive indolent lymphomas.

One key finding of this study was that WB-DWIBS/STIR had a high sensitivity and specificity for evaluating indolent lymphoma in whole-body regions. These results closely matched those of Mayerhoefer et al. [18]. Notably, the present study showed that there was an advantage in using WB-DWIBS/STIR over ^18^F-FDG-PET/CT in terms of detecting extra-nodal lesions. Our data shows that WB-DWIBS/STIR had a higher sensitivity (about 15%) compared to ^18^F-FDG-PET/CT for extranodal sites. For nodal regions, WB-DWIBS/STIR showed a similar or slightly higher sensitivity compared to ^18^F-FDG-PET/CT. In detail, on ^18^F-FDG-PET/CT, both readers missed five of the 23 extranodal sites in the present study. However, on WB-MRI, both readers missed only one lesion out of the 23 extranodal lesions. It can be reasonably explained by the fact that WB-MRI, particularly DWI, produces image contrast based on the degree of cellularity of the lymphoma, although indolent non-Hodgkin’s lymphoma has low glucose metabolism. Thus, our study demonstrates that WB-MRI is more useful for the detection of positive extranodal sites in indolent lymphoma compared to ^18^F-FDG-PET/CT, although the number of extranodal sites may be insufficient to robustly conclude this result.

In the present study, WB-DWIBS/STIR showed limitations for assessing lymphoma involvement in the spleen, gastrointestinal tract, and some thoracic lymph nodes. The WB-DWIBS/STIR findings disagreed with the reference standard for the pretherapeutic staging in one of the 30 patients with indolent lymphoma, with two observers (observers 1 and 2) over-staging one patient with CLL/SLL from III to IIIS. The reason was that splenomegaly (> 13 cm in maximal size) was used as a diagnostic criterion for splenic involvement of lymphoma. Cheson et al. [2] recommended a cutoff of 13 cm for the diagnosis of splenomegaly; however, the size of the spleen is affected by the height and body size [34]. In addition, 30% of normal-sized spleens can have tumor infiltration, and splenomegaly may also occur without tumor infiltration [35, 36]. Moreover, the splenomegaly evaluation criteria are controversial, although the splenic index scores calculated from the maximum craniocaudal height, transverse thickness, and anteroposterior length show a relatively high sensitivity and specificity [35, 37]. However, as WB-DWIBS/STIR was presented only in the coronal plane, it limited the accurate evaluation of splenomegaly. Therefore, there is a need for the exact diagnostic criteria for spleen involvement of lymphoma on WB-DWIBS/STIR. Two observers missed one of the two gastric lesions on WB-DWIBS/STIR. Similar to previous studies [18, 38, 39], WB-MRI seemed to be limited in evaluation of the organs subject to movement, such as the gastrointestinal tract. However, the number of extranodal sites in the present study was insufficient to draw clear conclusions from this result, and additional follow-up studies are required. For thoracic nodal regions (i.e., hilum, internal mammary region, cardiophrenic angle, and intercostal area), WB-DWIBS/STIR showed relatively less agreement with the reference standard compared to other nodal regions. This can be explained by the fact that cardiac pulsation and respiratory breathing motion can affect the MRI signal and lesion detection in the mediastinum [19]. Therefore, knowing the limitations of WB-DWIBS/STIR for assessing lymphoma involvement in these body regions may be important for clinicians and radiologists in their determination of the precise stage of the disease.

There are several limitations to this study. First, because of the relatively high incidence of follicular lymphomas and MALTomas, the numbers of indolent lymphoma subtypes were dissimilar, although the included numbers are a reflection of the true incidence of each subtype of indolent lymphoma treated in our hospital. Therefore, our evaluation of the usefulness of WB-DWIBS/STIR for indolent lymphomas, such as small lymphocytic or nodal marginal zone lymphomas, is limited. Second, correlations with histopathology were not performed for all nodal lesions included in this study. However, histological examination of all nodal lesions is practically impossible and has ethical concerns. To overcome these limitations, we used the multimodality and multidisciplinary consensus review as the reference standard for lesions discordant between WB-DWIBS/STIR and ^18^F-FDG-PET/CT. Third, our study included one patient with a 945-day interval between the biopsy and the WB-DWIBS/STIR scan. That patient was confirmed with MALToma using a lung biopsy and was observed on follow-up CT because of refusal to undergo treatment. Although the time interval between the biopsy and WB-DWIBS/STIR was long, its influence on the study may have been insignificant considering that the lesion did not show any change on follow-up CT and that the patient did not receive any treatment. Fourth, most extranodal lesions examined in our study had sufficient tumor volume to be visible on contrast-enhanced or follow-up CT, so there may be limitations in the generalization of our results to small extranodal lesions that are difficult to visualize. Finally, image distortion and signal loss artifacts, which are generally indicated as disadvantages of DWI, were not included in the image evaluation. These may limit the diagnostic performance in clinical practice.

## Conclusions

WB-DWIBS/STIR is an effective modality for the pretherapeutic staging of indolent lymphoma, and it has benefits when evaluating extranodal lesions, compared with ^18^F-FDG-PET/CT.

## Data Availability

Please contact the corresponding author regarding any requests for the data used or analyzed in this study.

## Abbreviations

WB-DWIBS/STIR: Whole-body diffusion-weighted imaging with background body signal suppression and T2-weighted short-tau inversion recovery MRI
WB-MRI: Whole-body magnetic resonance imaging
DWI: Diffusion-weighted imaging
^18^F-FDG-PET/CT: 18F-fluorodeoxyglucose-positron emission tomography/computed tomography
CI: Confidence interval
ADC: Apparent diffusion coefficient
CT: Computed tomography
CLL/SLL: Chronic lymphocytic leukemia/small lymphocytic lymphoma
MALToma: Extranodal marginal zone lymphoma of mucosa-associated lymphoid tissue
